# Supplementary material for: AMMI analysis of elite bread wheat (Triticum aestivum L.) selections for genotype by environment interaction and stability of grain yield in Southern Ethiopia
Source: PLoS One. 2025 Jan 30;20(1):e0318559. doi: 10.1371/journal.pone.0318559 (PMC11781717; doi:10.1371/journal.pone.0318559)
Supplement: S1 Table — (PDF) [file pone.0318559.s001.pdf]

## Supplementary information

## S1 Table. Summary of mean squares of 16 traits among 11 bread wheat genotypes tested at each location

Boloso Sore location

| Traits | Df | DH | DM | GFP | PH | NTTP | ENT | NSS | NKS | SL | TKW | HI | AGBM | LR | YR | SR | GY |
|---|---|---|---|---|---|---|---|---|---|---|---|---|---|---|---|---|---|
| Replication | 2 | 16.39 | 113.67 | 78.82 | 20.44 | 22.32 | 22.725 | 9.98 | 24.55 | 2.58 | 6.84 | 22.08 | 1.11 | 13.83 | 0.19 | 0.19 | 0.48 |
| Genotypes | 10 | 39.23** | 61.9* | 35.07* | 357.16** | 5.80$^{ns}$ | 5.56$^{ns}$ | 6.75* | 207.69** | 1.85* | 16.67** | 37.4** | 3.52* | 7.69$^{ns}$ | 4.74$^{ns}$ | 0.34$^{ns}$ | 0.52* |
| Error | 20 | 2.69 | 12.63 | 11.12 | 17.20 | 8.16 | 7.84 | 2.29 | 20.38 | 0.42 | 1.64 | 4.98 | 0.96 | 6.95 | 3.94 | 0.4 | 0.1 |
| CV% | | 2.6 | 3.5 | 8.8 | 3.8 | 35.8 | 36.9 | 8.1 | 10.9 | 6.9 | 3.7 | 8.0 | 10.0 | 45.8 | 22.6 | 12.2 | 11.8 |

Damot Gale location

| Traits | Df | DH | DM | GFP | PH | NTTP | ENT | NSS | NKS | SL | TKW | HI | AGBM | LR | YR | SR | GY |
|---|---|---|---|---|---|---|---|---|---|---|---|---|---|---|---|---|---|
| Replication | 2 | 0.64 | 1.85 | 3.27 | 61.02 | 8.99 | 7.58 | 5.18 | 111.11 | 0.48 | 5.55 | 21.14 | 11.48 | 0.19 | 7.01 | 1.338 | 0.05 |
| Genotypes | 10 | 20.62** | 16.66* | 20.67** | 516.14** | 6.56$^{ns}$ | 5.67$^{ns}$ | 9.29* | 104.76* | 3.27* | 112.02** | 50.21** | 7.89* | 4.09$^{ns}$ | 206.7** | 1.82$^{ns}$ | 0.31** |
| Error | 20 | 1.04 | 3.48 | 2.74 | 45.40 | 5.06 | 4.95 | 2.82 | 33.67 | 0.92 | 3.99 | 6.08 | 2.24 | 2.27 | 10.97 | 0.91 | 0.03 |
| CV% | | 1.5 | 1.5 | 3.0 | 6.2 | 28.2 | 29.2 | 9.2 | 13.9 | 9.3 | 4.6 | 7.4 | 14.0 | 25.2 | 29.9 | 17.7 | 4.6 |

Humbo location

| Traits | Df | DH | DM | GFP | PH | NTTP | ENT | NSS | NKS | SL | TKW | HI | AGBM | LR | YR | SR | GY |
|---|---|---|---|---|---|---|---|---|---|---|---|---|---|---|---|---|---|
| Replication | 2 | 3.67 | 1.09 | 8.76 | 8.17 | 1.54 | 0.48 | 1.35 | 7.80 | 1.93 | 8.85 | 4.85 | 0.05 | 3.98 | 2.46 | 2.46 | 0.02 |
| Genotypes | 10 | 141.94** | 75.96* | 55.87* | 85.61* | 4.61* | 1.59* | 13.20** | 235.09** | 2.77* | 22.28** | 100.35** | 1.09$^{ns}$ | 9.92* | 6.40* | 7.27$^{ns}$ | 0.17* |
| Error | 20 | 2.567 | 17.36 | 21.89 | 31.34 | 0.95 | 0.57 | 1.92 | 21.15 | 0.68 | 3.24 | 4.70 | 0.73 | 3.56 | 2.25 | 3.3 | 0.04 |
| CV% | | 2.5 | 4.4 | 15.3 | 9.3 | 14.9 | 16.0 | 8.4 | 14.0 | 10.0 | 7.3 | 9.2 | 32.7 | 23.1 | 15.6 | 31.5 | 31.1 |

Kokate location

| Traits | Df | DH | DM | GFP | PH | NTTP | ENT | NSS | NKS | SL | TKW | HI | AGBM | LR | YR | SR | GY |
|---|---|---|---|---|---|---|---|---|---|---|---|---|---|---|---|---|---|
| Replication | 2 | 28.45 | 17.49 | 1.48 | 47.18 | 0.47 | 0.13 | 3.94 | 62.04 | 0.85 | 9.6 | 36.18 | 12.6 | 0.76 | 12.69 | 0.76 | 1.89 |
| Genotypes | 10 | 72.30* | 12.93* | 75.90* | 208.75** | 8.78ns | 8.10* | 9.39** | 167.47** | 2.38* | 59.73** | 19.77* | 6.28* | 1.40ns | 46.36** | 0.76ns | 0.78** |
| Error | 20 | 22.19 | 2.652 | 26.55 | 17.26 | 3.76 | 2.51 | 1.14 | 15.45 | 0.56 | 2.1 | 6.06 | 0.15 | 1.17 | 7.27 | 0.76 | 0.07 |
| CV% | | 6.4 | 1.3 | 9.7 | 4.3 | 24.1 | 22.8 | 6.1 | 8.6 | 7.3 | 3.9 | 13.8 | 15.3 | 20.1 | 34.6 | 16.9 | 15.3 |

Where: - ** - Significant at P<0.01, * - Significant at P<0.05, and ns - the non-significant, number in parenthesis represented the degree of freedom for the respective source of variation. DH = days to heading, DM = days to maturity, GFP=grain filling period, PH = plant height (cm), SL= spike length (cm), NKS = number of kernels spike$^{-1}$, NSS=spiklets spike$^{-1}$, NTTP=number of total tillers plant$^{-1}$, ENT =effective number of tillers plant-1, SR=stem rust(scale), LR=leaf rust, YR=yellowSilase1221@lili rust (scale), GY=grain yield (t/ha), ABM=biomass yield (t/ha), HI=harvest index, TKW=thousand kernel weight (g).
