# Supplementary material for: AMMI analysis of elite bread wheat (Triticum aestivum L.) selections for genotype by environment interaction and stability of grain yield in Southern Ethiopia
Source: PLoS One. 2025 Jan 30;20(1):e0318559. doi: 10.1371/journal.pone.0318559 (PMC11781717; doi:10.1371/journal.pone.0318559)
Supplement: S2 Table — (PDF) [file pone.0318559.s002.pdf]

S2 Table. Mean values for different agronomic traits of the 11 genotypes at each environment in 2022.

Environment	Genotypes	DH	DM	GFP	PH	NTTP	ENTP	NSS	NKS	SL	TKW	HI	AGBM	LR	YR	SR	GY
Boloso Sore	226930 (G1)	64.7^{bc}	104.7^{ab}	40.0^{abc}	132^{a}	7.53	7.40	17.40^{b}	42.40^{bc}	8.83^{cd}	37.83^{a}	23.53^{c}	12.13a	5.83	7.50	5.00	2.84^{b}
	227248 (G2)	60.33^{e}	103.0^{b}	42.67^{ab}	97.4^{de}	8.07	7.70	17.60^{b}	39.40^{bc}	8.44^{d}	34.83^{bc}	27.94^{b}	9.73 bc	7.50	11.67	5.83	2.72^{bc}
	238133 (G3)	65.00^{bc}	103.3^{b}	38.33^{abcd}	114.8^{b}	9.60	9.27	17.27^{b}	36.67^{bc}	9.567^{bcd}	31.50^{ef}	22.30^{c}	9.79^{bc}	10.0	7.50	5.83	2.19^{c}
	238139 (G4)	69.00^{a}	110.33^{a}	41.33^{abc}	125.2^{a}	8.00	7.40	17.65^{b}	43.43^{b}	8.507^{d}	32.33^{def}	25.36^{bc}	10.79^{ab}	5.00	7.50	5.00	2.73^{bc}
	238498 (G5)	64.33^{bc}	100.33^{bc}	36.00^{cd}	108.9^{bc}	8.07	7.73	19.27^{ab}	39.60^{b}c	9.07^{bcd}	34.17^{bcd}	28.60^{b}	9.58^{bc}	5.00	8.33	5.00	2.74^{bc}
	238507 (G6)	66.67^{ab}	103.33^{b}	36.67^{bcd}	104.2^{cd}	10.93	10.40	20.93^{a}	40.87^{bc}	9.76^{bc}	33.17^{cde}	28.42^{b}	8.04 c	5.00	9.17	5.00	2.28^{bc}
	238525 (G7)	66.00^{bc}	101.67^{bc}	35.67^{cd}	105.8^{c}	7.40	7.07	20.67^{a}	41.60^{bc}	9.47^{bcd}	30.67^{f}	25.88^{bc}	8.95^{bc}	5.00	9.17	5.00	2.33^{bc}
	238543 (G8)	63.33^{cd}	96.00^{c}	32.67^{d}	104.4^{cd}	7.67	7.53	19.20^{ab}	35.60^{bc}	10.28^{ab}	34.83^{bc}	29.69^{b}	9.743^{bc}	5.00	8.33	5.00	2.89^{b}
	238871 (G9)	59.33^{ef}	95.67^{c}	36.33^{bcd}	108.9^{bc}	7.53	7.13	18.47^{ab}	35.93^{bc}	9.793^{bc}	35.33^{bc}	29.21^{b}	8.903^{bc}	5.00	9.167	5.00	2.625^{bc}
	238873 (G10)	56.67^{f}	95.67^{c}	35.67^{cd}	111.0^{bc}	7.60	6.87	17.27^{b}	33.73^{c}	9.18^{bcd}	36.00^{ab}	28.98^{b}	9.537^{bc}	5.00	10.00	5.00	2.765^{bc}
	Alidoro (G11)	61.00^{de}	104.67^{ab}	43.67^{a}	95.6^{e}	5.33	5.00	20.93^{a}	64.53a	11.100^{a}	37.83^{a}	35.45^{a}	10.54^{ab}	5.00	8.33	5.00	3.74^{a}
	Range	56.67-69	95.67 – 110.33	32.67 – 43.67	95.6 – 132.0	5.33 – 10.93	5.00-10.40	17.27 - 20.93	33.73-64.53	8.44-11.10	30.67 - 37.83	22.3-35.45	8.038-12.13	5.0-10.00	7.5-11.67	5.00 – 5.83	2.19 - 3.74
	Mean	63.3	101.7	38.09	109.8	7.98	7.59	18.79	41.25	9.45	34.41	27.76	9.79	5.76	8.79	5.15	2.71
	LSD 5%	2.8	6.05	5.68	7.06	4.87	4.77	2.58	7.69	1.11	2.18	3.8	1.67	4.49	3.38	1.07	0.55
	CV%	2.6	3.5	8.8	3.8	35.8	36.9	8.1	10.9	6.9	3.7	8	10	45.8	22.6	12.2	11.8
Damot Gale	226930 (G1)	68.67^{d}	127.0^{b}	57.67^{b}	134.8^{a}	6.90	6.47	17.47^{bcde}	43.87^{b}	9.71^{bcd}	46.83^{abc}	30.92^{c}	11.78^{a}	8.33	11.67^{bcd}	5.00	3.61^{ab}
	227248 (G2)	63.33^{e}	125.0^{b}	61.67^{a}	85.4^{f}	6.60	6.13	14.73^{ce}	33.07^{b}	8.50^{d}	30.50^{e}	22.89^{d}	6.32^{b}	6.67	33.33^{a}	5.00	1.42^{c}

	238133 (G3)	69.33^{cd}	125.7^{b}	56.33^{bc}	122.1^{b}	7.40	7.20	17.87^{bc}	39.13^{b}	9.58^{bcd}	37.33^{d}	32.13^{bc}	10.74^{a}	5.00	11.67^{bcd}	7.50	3.44^{ab}
	238139 (G4)	73.33^{a}	131.0^{a}	57.67^{b}	117.4^{bc}	9.53	9.00	16.60^{bcde}	40.27^{b}	9.18^{cd}	35.83^{d}	31.70^{c}	9.58^{a}	5.83	12.50^{bc}	5.83	3.08^{b}
	238498 (G5)	71.00^{bc}	125.0^{b}	54.00^{cde}	106.2^{cde}	7.53	7.40	18.40^{b}	36.67^{b}	10.23^{abcd}	46.00^{abc}	34.84^{abc}	11.4^{a}	6.67	5.83^{de}	5.83	3.91^{ab}
	238507 (G6)	71.33^{b}	124.3^{bc}	53.00^{de}	112.4^{bcd}	8.33	8.13	17.87^{bcd}	42.73^{b}	10.71^{abc}	44.00^{c}	36.83^{ab}	10.69^{a}	5.00	5.83^{de}	5.00	3.96^{ab}
	238525 (G7)	71.33^{b}	127.0^{b}	55.67^{bcd}	108.1^{cde}	7.00	6.73	18.53^{b}	39.07^{b}	10.25^{abcd}	43.17^{c}	35.03^{abc}	10.65a	5.0	7.50^{cde}	5.00	3.74^{ab}
	238543 (G8)	71.00^{bc}	126.0^{b}	55.00^{bcde}	103.8^{de}	9.33	8.93	18.67^{b}	42.60^{b}	11.39^{ab}	48.17^{ab}	35.51^{abc}	11.73a	7.50	5.00^{e}	5.00	4.15^{ab}
	238871(G9)	69.33^{cd}	121.3^{c}	52.00 e	103.5^{de}	7.467	7.07	19.67^{ab}	43.40^{b}	10.25^{abcd}	49.17^{a}	37.76^{a}	10.86^{a}	5.00	5.83^{de}	5.00	4.1^{ab}
	238873 (G10)	68.67^{d}	124.7^{bc}	56.00^{bcd}	113.4^{bcd}	11.20	10.53	18.60^{b}	43.33^{b}	11.73^{a}	49.33^{a}	35.11^{abc}	12.37^{a}	5.83	5.83^{de}	5.00	4.33^{a}
	Alidoro (G11)	67.67^{d}	124.7^{bc}	57.0^{bc}	96.3^{ef}	6.47	6.33	21.80^{a}	56.6^{a}	11.75^{a}	45.17^{bc}	35.22^{abc}	11.29a	5.00	16.67^{b}	5.00	3.97^{ab}
	Range	63.33 – 73.33	121.3 – 131.0	52.00 – 61.67	85.4 – 134.8	6.467 – 11.20	6.133 – 10.533	14.73 – 21.80	33.07 – 56.60	8.50 – 11.75	30.50 – 49.33	22.89 – 37.76	6.32 – 12.37	5.00 – 8.333	5.00 – 33.33	5.000 – 7.50	1.42 – 4.33
	Mean	69.55	125.61	56	109.4	7.98	7.63	18.2	41.9	10.3	43.23	33.45	10.67	5.98	11.06	5.38	3.61
	LSD 5%	1.73	3.18	2.82	11.48	3.83	3.79	2.86	9.88	1.629	3.4	4.2	2.55	2.57	5.64	1.62	0.95
	CV%	1.5	1.5	3	6.2	28.2	29.2	9.2	13.3	9.3	4.6	7.4	14	25.2	29.9	17.7	4.6
Humbo	226930 (G1)	57.00^{cd}	89.33^{c}	32.33^{abc}	67.89^{a}	5.467^{cd}	4.20^{bcd}	17.07^{ab}	39.4^{b}	8.287^{abcde}	27.33^{a}	21.83^{de}	2.73	5.83^{c}	8.33^{bc}	6.67	0.61^{bcd}
	227248 (G2)	59.00^{bc}	92.00^{bc}	33.00^{abc}	49.54^{d}	4.80^{d}	3.60^{d}	14.13^{cd}	31.93^{bc}	6.95^{e}	22.33^{bc}	29.90^{b}	1.37	5.83^{c}	9.17^{abc}	6.67	0.41^{cd}
	238133 (G3)	59.00^{bc}	98.67^{ab}	39.67^{a}	63.71^{abc}	7.60^{ab}	5.87^{a}	13.33^{d}	29.40^{c}	7.23^{e}	27.83^{a}	26.58^{bc}	3.39	10.00^{ab}	10.00^{ab}	10.0	0.91^{ab}
	238139 (G4)	72.33^{a}	104.00^{a}	31.67^{abc}	63.05^{abc}	8.13^{a}	4.93^{abcd}	13.27^{d}	30.33^{c}	7.56^{cde}	20.00^{c}	14.96^{g}	2.42	7.50^{bc}	6.667^{c}	5.00	0.34^{d}
	238498 (G5)	71.67^{a}	97.33^{ab}	25.67^{bc}	59.23^{abcd}	6.07^{bcd}	3.87^{cd}	17.33^{ab}	29.8^{c}	8.07^{bcde}	23.83^{b}	19.82^{ef}	2.08	9.17^{abc}	10.00^{ab}	5.00	0.42^{cd}
	238507 (G6)	70.00^{a}	95.00^{bc}	25.00^{c}	53.59^{cd}	6.13^{bcd}	4.67^{abcd}	16.20^{bc}	25.93^{c}	7.43^{de}	22.17^{bc}	17.20^{fg}	2.35	9.17^{abc}	10.00^{ab}	5.00	0.4^{cd}

	238525 (G7)	71.67^{a}	98.33^{ab}	26.67^{bc}	61.01^{abc}	7.73^{ab}	5.53^{ab}	17.47^{ab}	28.87^{c}	9.00^{abcd}	23.17^{bc}	20.13^{ef}	2.29	9.17^{abc}	11.67^{a}	5.00	0.46^{cd}
	238543 (G8)	70.33^{a}	99.00^{ab}	28.67^{bc}	56.77^{bcd}	7.53^{ab}	4.67^{abcd}	17.27^{ab}	29.40^{c}	9.02^{abc}	23.67^{b}	21.52^{de}	2.74	11.67^{a}	10.00^{ab}	5.00	0.6^{bcd}
	238871(G9)	60.67^{b}	89.00^{c}	28.33^{bc}	58.65^{abcd}	6.93^{abc}	5.40^{ab}	16.53^{bc}	27.7^{c}	8.380^{abcde}	27.67^{a}	24.49^{cd}	3.51	7.50^{bc}	11.67^{a}	5.00	0.86^{ab}
	238873 (G10)	60.33^{b}	91.33^{bc}	31.00^{abc}	60.46^{abc}	7.067^{abc}	5.267^{abc}	18.80^{ab}	32.47^{bc}	9.573^{ab}	25.50^{ab}	26.63^{bc}	2.83	7.50^{bc}	10.00^{ab}	5.00	0.76^{abc}
	Alidoro (G11)	54.67^{d}	89.00^{c}	34.33^{ab}	65.96^{ab}	4.47^{d}	4.20^{bcd}	19.67^{a}	57.47^{a}	9.80^{a}	27.83^{a}	34.86^{a}	2.96	6.67^{bc}	8.33^{bc}	5.00	1.04^{a}
	Range	54.67 - 72.33	89.00 - 104.0	25.0-39.67	49.54 - 67.89	4.8-8.13	3.6-5.867	13.27 - 19.67	25.93-57.47	6.95-9.80	20.00 - 27.83	14.96 - 34.86	1.37-3.51	5.83 - 11.67	6.67-11.67	5.0-10.0	0.34 - 1.04
	Mean	64.24	94.82	30.58	59.99	6.54	4.75	16.46	32.92	8.3	24.67	23.45	2.61	8.18	9.62	5.76	0.617
	LSD 5%	2.73	7.1	7.97	9.54	1.66	1.29	2.36	7.83	1.41	3.07	3.69	1.45	3.21	2.56	3.09	0.33
	CV%	2.5	4.4	15.3	9.3	14.9	16	8.4	14	10	7.3	9.2	32.7	23.1	15.6	31.5	31.1
Kokate	226930 (G1)	72.33^{ab}	128.7^{ab}	56.33^{ab}	106.29^{a}	6.80	5.00cd	17.07^{bcd}	43.67^{cd}	9.72^{a}	38.67^{b}	25.76^{d}	8.475^{b}	5.00	13.33^{ab}	5.00	2.19^{b}
	227248 (G2)	67.00^{bc}	127.0^{abc}	60.00^{a}	77.72^{d}	6.067	5.133^{cd}	14.40^{e}	38.80^{d}	8.06^{b}	29.83^{f}	30.01^{abcd}	4.76^{c}	5.00	16.67^{a}	5.00	1.26^{c}
	238133 (G3)	72.33^{ab}	126.3^{bc}	54.00^{g}	105.58^{a}	8.93	7.867^{abc}	15.40^{de}	42.13^{cd}	9.91^{a}	33.17^{de}	30.59^{abc}	7.96^{b}	5.00	5.83^{c}	6.67	2.44^{b}
	238139 (G4)	79.67^{a}	129.7^{a}	50.00^{b}	103.90^{ab}	10.60	9.07^{ab}	15.33^{de}	47.33^{bc}	9.72^{a}	31.00^{ef}	27.43^{bcd}	8.64^{b}	5.00	6.67^{c}	5.00	2.41^{b}
	238498 (G5)	78.00^{a}	127.0^{abc}	49.0^{b}	98.91^{ab}	8.80	7.733^{abc}	18.93^{ab}	51.4^{b}	10.46^{a}	38.17^{b}	30.38^{abcd}	8.23^{b}	5.83	5.83^{c}	5.00	2.53^{b}
	238507 (G6)	77.33^{a}	124.7^{cd}	47.33^{b}	99.75^{ab}	7.07	6.200^{bcd}	19.60^{a}	48.13^{bc}	10.67^{a}	37.50^{bc}	29.30^{bcd}	8.88^{ab}	5.00	5.00^{c}	5.00	2.61^{b}
	238525 (G7)	76.67^{a}	126.0^{bc}	49.33^{b}	96.31^{b}	10.33	9.533^{a}	19.40^{a}	42.27^{cd}	10.81^{a}	35.50^{cd}	31.40^{ab}	8.45^{b}	5.00	5.00^{c}	5.00	2.65^{b}
	238543 (G8)	76.00^{a}	125.0^{c}	49.00^{b}	96.96^{b}	8.80	7.667^{abc}	16.87^{cd}	38.80^{d}	11.06^{a}	39.00^{b}	30.01^{abcd}	8.94^{b}	6.67	7.50^{c}	5.00	2.635^{b}
	238871(G9)	72.33^{ab}	122.0^{d}	49.67^{b}	98.54^{ab}	7.60	6.733^{abcd}	17.93^{abc}	39.47^{d}	11.14^{a}	43.83^{a}	31.76^{ab}	10.89^{a}	6.67	5.00^{c}	5.00	3.44^{a}
	238873 (G10)	72.00^{ab}	127.7^{abc}	55.67^{ab}	97.04^{b}	8.80	6.93^{abcd}	18.53^{abc}	44.07^{cd}	10.85^{a}	42.67^{a}	32.21^{ab}	8.24^{b}	5.00	5.00^{c}	5.00	2.67^{b}

Alidoro (G11)	63.33^{c}	125.7^{bc}	62.33^{a}	86.87^{c}	5.067	4.47^{d}	18.33^{abc}	64.47^{a}	10.71^{a}	39.83^{b}	34.16^{a}	7.48^{b}	5.00	10.00^{bc}	5.00	2.56^{b}
Range	63.33-79.67	122.0-129.7	47.33-62.33	77.72-106.29	5.07-10.60	4.47-9.53	14.4-19.6	38.8-64.47	8.06-11.14	29.83-43.83	25.76-34.16	4.755-10.89	5.0-6.67	5.0-16.67	5.00–6.67	1.26–3.44
Mean	73.36	126.3	52.97	97.08	8.04	6.94	17.44	45.5	10.29	37.2	29.94	8.27	5.38	7.8	5.15	2.49
LSD 5%	8.02	2.77	8.78	7.08	3.3	2.7	1.82	6.69	1.27	2.47	4.19	1.92	1.85	4.59	1.48	0.65
CV%	6.4	1.3	9.7	4.3	24.1	22.8	6.1	8.6	7.3	3.9	8.2	13.6	20.1	34.6	16.9	15.3

Means with the same letters are not statistically significant. Where, DH = days to heading, DM = days to maturity, GFP=grain filling period, PH = plant height (cm), SL= spike length (cm), NKS = number of seeds spike^{-1}, NSS=spiklets spike^{-1}, NTTP=number of total tillers plant^{-1}, ENTP =effective number of tillers plant-1, SR=stem rust(scale), LR=leaf rust, YR=yellow rust (scale), GY=grain yield (t/ha), ABM=biomass yield (t/ha), HI=harvest index, TKW=thousand seed weight (g).
