# Supplementary material for: AMMI analysis of elite bread wheat (Triticum aestivum L.) selections for genotype by environment interaction and stability of grain yield in Southern Ethiopia
Source: PLoS One. 2025 Jan 30;20(1):e0318559. doi: 10.1371/journal.pone.0318559 (PMC11781717; doi:10.1371/journal.pone.0318559)
Supplement: S3 Table — (PDF) [file pone.0318559.s003.pdf]

S3 Table. Mean values for different agronomic traits of the 11 genotypes across environment in 2022

Genotypes	DH	DM	GFP	PH	NTTP	ENTP	NSS	NKS	SL	TKW	HI	AGBM	LR	YR	SR	GY
226930 (G1)	65.67^{c}	112.4^{bc}	46.58	110.23^{a}	6.68	5.77	17.25^{c}	42.33^{b}	9.14	37.67^{ab}	25.51^{e}	8.78^{a}	6.25^{abc}	10.21^{bc}	5.42	2.31^{cd}
227248 (G2)	62.42^{de}	111.8^{bc}	49.33	77.51^{f}	6.38	5.65	15.22^{d}	35.80^{c}	7.99	29.38^{g}	26.78^{de}	5.544^{c}	6.25^{abc}	17.71^{a}	5.63	1.45^{e}
238133 (G3)	66.42^{c}	113.5^{b}	47.08	101.54^{b}	8.38	7.55	15.97^{d}	36.68^{c}	9.07	32.46^{f}	27.9^{cd}	7.97^{ab}	7.50^{ab}	8.75^{bcd}	7.50	2.24^{cd}
238139 (G4)	73.58^{a}	118.8^{a}	45.17	102.39^{b}	9.07	7.60	15.71^{d}	40.34^{bc}	8.76	29.79^{g}	24.86^{e}	7.86^{ab}	5.83^{bc}	8.33^{cd}	5.21	2.14^{d}
238498 (G5)	71.25^{ab}	112.4^{bc}	41.17	93.31^{cd}	7.62	6.68	18.48^{bc}	39.37^{bc}	9.46	35.54^{cd}	28.41^{cd}	7.82^{ab}	6.67^{abc}	7.500^{d}	5.21	2.4^{bcd}
238507 (G6)	71.33^{ab}	111.8^{bc}	40.50	92.47^{cd}	8.12	7.35	18.65^{b}	39.42^{bc}	9.64	34.21^{de}	27.94^{cd}	7.49^{b}	6.04^{abc}	7.50^{d}	5.00	2.31^{cd}
238525 (G7)	71.42^{ab}	113.2^{b}	41.83	92.81^{cd}	8.12	7.217	19.02^{ab}	37.95^{bc}	9.88	33.12^{ef}	28.11^{cd}	7.58^{ab}	6.04^{abc}	8.33^{cd}	5.00	2.3^{cd}
238543 (G8)	70.17^{b}	111.5^{bc}	41.33	90.49^{d}	8.33	7.20	18.00^{bc}	36.60^{c}	10.44	36.42^{bc}	29.18^{bc}	8.29^{ab}	7.71^{a}	7.71^{d}	5.00	2.57^{abc}
238871(G9)	65.42^{c}	107.0^{d}	41.58	92.39^{cd}	7.38	6.58	18.15^{bc}	36.63^{c}	9.89	39.00^{a}	30.81^{b}	8.54^{ab}	6.04^{abc}	7.92^{d}	5.00	2.75^{ab}
238873 (G10)	64.42^{cd}	109.8^{c}	44.58	95.46^{c}	8.5	7.40	18.30^{bc}	38.40^{bc}	10.33	38.38^{a}	30.73^{b}	8.24^{ab}	5.83^{bc}	7.71^{d}	5.00	2.63^{abc}
Alidoro (G11)	61.67^{e}	111.0^{bc}	49.33	86.17^{e}	5.33	5.00	20.18^{a}	60.77^{a}	10.84	37.67^{ab}	34.92^{a}	8.07^{ab}	5.42^{c}	10.83^{b}	5.00	2.825^{a}
Range	61.67-73.58	107.0-118.8	40.50-49.33	77.5-110.23	5.33-9.07	5.000-7.60	15.22-20.18	35.80-60.77	7.99-10.84	29.38-39.00	24.86-34.92	5.544-8.78	5.42-7.71	7.500-17.71	5.00-7.50	1.45-2.83
Mean	67.61	112.1	44.4	94.07	7.63	6.73	17.72	40.39	9.586	34.88	28.65	7.83	6.33	9.32	5.36	2.357
LSD 5%	2.249	2.696	3.201	4.28	1.8	1.697	1.187	4.0	0.692	1.403	1.999	1.029	1.52	1.994	0.95	0.343
CV%	4.1	3	8.9	5.6	29.1	31.1	8.3	12.2	8.9	5	13.5	16.2	8.9	10.8	21.7	17.9

Means with the same letters are not statistically significant. Where, DH = days to heading, DM = days to maturity, GFP=grain filling period, PH = plant height (cm), SL= spike length (cm), NKS = number of kernels spike^{-1}, NSS=spikelets spike^{-1}, NTTP=number of total tillers plant^{-1}, ENTP =effective number of tillers plant-1, SR=stem rust(scale), LR=leaf rust, YR=yellow rust (scale), GY=grain yield (t/ha), ABM=biomass yield (t/ha), HI=harvest index, TKW=thousand kernel weight (g).
