# Supplementary material for: AMMI analysis of elite bread wheat (Triticum aestivum L.) selections for genotype by environment interaction and stability of grain yield in Southern Ethiopia
Source: PLoS One. 2025 Jan 30;20(1):e0318559. doi: 10.1371/journal.pone.0318559 (PMC11781717; doi:10.1371/journal.pone.0318559)
Supplement: Attachment — Submitted filename: PONE-D-24-31553_reviewer1.pdf [file pone.0318559.s004.pdf]

Review Plos one

AMMI Analysis of Elite Bread Wheat (Triticum aestivum L.) Selections for Genotype byEnvironment Interaction and Stability of Grain Yield in Southern Ethiopia

- The authors didnt shows the aim of the research in the abstract section.
- For citation format, authors must re-read the author guidelines.
- Inconsistent in the units used (grain yield (t/ha), 2.67 t ha-1, and 2.51 ton ha-1), check in all sections for all units.
- It would be interesting to conduct a correlation analysis or path analysis between environmental factors (temperature, rainfall, altitudes, and soil conditions) with yield and yield attributes.
- It needs to be explained that AMMI, ASV, YSI, and GGE Biplot are used to identify stability. And why use these analyses? There are many other stability analyses that can be used, such as combined stability analysis (parametric and non-parametric). If combined between Visual Analysis (AMMI and GGE biplots) with parametric and non-parametric, it will be very interesting and more precise (authors can read and cite some of these articles: https://doi.org/10.1007/s10681-019-2386-5 and https://doi.org/https://doi.org/10.34044/j.anres.2022.56.4.10)
- There is no information on soil conditions at each location. Because soil fertility is one of the factors that affect crop yields.
- The subtitle "Mean Performance of the 11 selections for the 16 traits evaluated across locations" is confusing.
- Inconsistent subtitles, such as: "Analysis of variance" uses sequential numbers, but other subtitles do not use sequential numbers. In addition, the subtitles are also less descriptive of what will be discussed and are too general.
- In table 5, the numbers after the decimal point are inconsistent.
- There are several typos, such as "spikelets", in several sentences it is written as "spiklets"
- Inconsistent paragraph writing.
- It is necessary to add data for one more planting season in each location so that the influence of the genotypes by environment interactions is more visible.
- The discussion is not strong. How did the authors determine the best genotype (Stable and high yield) from all the analyses used?
- The use of the abbreviation of Genotype by environment interactions is inconsistent. In some parts it is written as GxL, but in other parts it is written as GxE
- The authors did not reveal the limits of genotype stability using AMMI and ASV. In AMMI Biplot, the radius of the ellipse should be displayed, which is one indicator of genotype stability.
- For mega environment analysis, the data used is still lacking.
- The authors do not disclose how they drew conclusions from all the analyses used.
- The conclusion section is not clear/ not concrete.
