# Supplementary material for: AMMI analysis of elite bread wheat (Triticum aestivum L.) selections for genotype by environment interaction and stability of grain yield in Southern Ethiopia
Source: PLoS One. 2025 Jan 30;20(1):e0318559. doi: 10.1371/journal.pone.0318559 (PMC11781717; doi:10.1371/journal.pone.0318559)
Supplement: Attachment — Submitted filename: Response to reviewers.2.docx [file pone.0318559.s007.docx]

**Authors Response to the Editor’s and Reviewers ’ Comments**

AMMI analysis of elite bread wheat (*Triticum aestivum* L.) selections for genotype by environment interaction and stability of grain yield in Southern Ethiopia

Ref. MS. NO. PONE-D-24-31553

PLOS ONE

**Editor’s comment:** Dear Dr. Gessese,

Thank you for submitting your manuscript to PLOS ONE. After careful consideration, we feel that it has merit but does not fully meet PLOS ONE’s publication criteria as it currently stands. Therefore, we invite you to submit a revised version of the manuscript that addresses the points raised during the review process. Please include the following items when submitting your revised manuscript:

- A rebuttal letter that responds to each point raised by the academic editor and reviewer(s). You should upload this letter as a separate file labeled 'Response to Reviewers'.
- A marked-up copy of your manuscript that highlights changes made to the original version. You should upload this as a separate file labeled 'Revised Manuscript with Track Changes'.
- An unmarked version of your revised paper without tracked changes. You should upload this as a separate file labeled 'Manuscript'.

Please review your reference list to ensure that it is complete and correct. If you have cited papers that have been retracted, please include the rationale for doing so in the manuscript text, or remove these references and replace them with relevant current references. Any changes to the reference list should be mentioned in the rebuttal letter that accompanies your revised manuscript. If you need to cite a retracted article, indicate the article’s retracted status in the References list and also include a citation and full reference for the retraction notice.

**Dear respected Professor Mehidi Rahimi (Academic editor), we thank you very much again for considering our work and recommended it for minor revision and further consideration. We have addressed all the suggestions, comments and made corrections to the mistakes requested by reviewer#3. In this rebuttal letter, we have provided point-by-point responses to all the comments provided by reviewer#3 for your reference and follow up of the review process. We prepared the new “manuscript” file following the guideline outlined by the editorial office so that it meets the journal’s standard. On behalf of all the authors, we request you to consider our manuscript for publication in your esteemed journal. Many thanks Sir for your valuable time and further consideration.**

**Reviewer #3:**

**Comment#1**: The authors have carefully addressed all the recommendations as suggested. However, a few recommendations need to be carried out before the manuscript (MS) is published. The description of the testing environments given under Section 2.1 is too descriptive and deviates from the scope of the journal. Therefore, I suggest that the authors add a single paragraph explaining the key information about the sites, as the other climatic and soil parameters are provided in the table.

**Author’s response: dear reviewer, we authors are very grateful for your unreserved effort to improve this manuscript and deliver your feedback. Based on your suggestion we made the correction as shown in the file ‘Revised manuscript with track changes’ within line numbers (L#) 134-171,**

**Comment#2**: In Section 2.3, the section number is missing for the data collection subheading. Additionally, the traits listed under this subheading are in italics; please change them to regular font.

**Author’s response: dear reviewer, thanks again for your comment we made the correction as shown in the file ‘Revised manuscript with track changes’ within line numbers (L#) 259-271.**

**Comment#3:** In the results under Sections 3.1 and 3.2, the traits can be mentioned in short form, with abbreviations explained in the materials section.

**Author’s response: dear reviewer, thanks again for your comment we made the correction as shown in the file ‘Revised manuscript with track changes’ within line numbers (L#) 351-354, 362-364, 371-372, 379, 383-385, 387-388, 394.**
